# DNA Damage and Augmented Oxidative Stress in Bone Marrow Mononuclear Cells from Angiotensin-Dependent Hypertensive Mice

**DOI:** 10.1155/2013/305202

**Published:** 2013-02-14

**Authors:** Bianca P. Campagnaro, Clarissa L. Tonini, Breno V. Nogueira, Dulce E. Casarini, Elisardo C. Vasquez, Silvana S. Meyrelles

**Affiliations:** ^1^Laboratory of Transgenes and Cardiovascular Control, Department of Physiological Sciences, Health Sciences Center, Federal University of Espirito Santo, Avenida Marechal Campos 1468, 29043-900 Vitoria, ES, Brazil; ^2^Department of Morphology, Health Sciences Center, Federal University of Espirito Santo, 29045-402 Vitoria, ES, Brazil; ^3^Department of Nephrology, Federal University of Sao Paulo (UNIFESP), 04021-001 Sao Paulo, SP, Brazil; ^4^College of Health Sciences, EMESCAM, 29043-900 Vitoria, ES, Brazil

## Abstract

It has been proposed that the nonhemodynamic effects of angiotensin II are important for the damage observed in the two-kidney, one-clip (2K1C) renovascular hypertension model. Much evidence confirms that angiotensin II is directly involved in NAD(P)H oxidase activation and consequent superoxide anion production, which can damage DNA. The current study was performed to examine the effects of angiotensin-II-dependent hypertension in bone marrow mononuclear cells (BM-MNC); dihydroethidium staining was used to assess reactive oxygen species (ROS) production, and the comet assay was used to assess DNA fragmentation in 2K1C hypertensive mice 14 days after renal artery clipping. In this study we demonstrated that 2K1C hypertensive mice have an elevated lymphocyte count, while undifferentiated BM-MNC counts were diminished. 2K1C mice also showed an augmented ROS production and marked BM-MNC DNA fragmentation. In conclusion, endogenous renin angiotensin system activation-induced arterial hypertension is characterized by excessive ROS production in BM-MNC, which might cause marked DNA damage.

## 1. Introduction

High blood pressure is commonly found in patients with chronic kidney disease and renovascular hypertension is a common form of secondary hypertension and frequently resistant to pharmacologic treatment [1]. In the two-kidney, one clip (2K1C) Goldblatt model, renovascular hypertension is induced by unilateral renal artery stenosis, which reduces renal perfusion of the clipped kidney and causes increased renin release and circulating angiotensin II (Ang II) [2]. Ang II, which is the main effector peptide of the renin-angiotensin system (RAS), has marked hemodynamic, cardiac, and renal effects, as previously observed by our laboratory in mice [2–5]. In addition, it also exerts tissue-specific responses as it can be locally synthesized [6–8]. Although it is controversial, the existence of a local bone marrow (BM) RAS has been demonstrated in rats [9]. Because the BM is a highly organized, complex organ, that is, the principal hematopoietic tissue in adults, locally BM-formed Ang II may be an autocrine or paracrine peptide that affects physiological and pathological hematopoiesis [10]. 

Studies have demonstrated that Ang II plays a role in oxidative stress development in the spontaneously hypertensive rat [11] and in the renovascular hypertensive rat [12]. Reactive oxygen species (ROS) play a crucial role in RAS signaling in BM cells [9, 13]. In addition, studies in experimental animals have shown that augmented ROS [14–16], particularly superoxide (•O_2_^−^) [17–20], can interact with DNA, which results in oxidative damage and DNA fragmentation-mediated cellular injury [21]. 

Taken together, this evidence strongly supports the importance of the 2K1C murine experimental model to investigate the influence of hypertension on DNA damage. Therefore, in the present study, we tested the hypothesis that 2K1C-mediated hypertension increases ROS production and induces DNA damage in murine BM mononuclear cells (MNC). 

## 2. Material and Methods

### 2.1. Animals

Experiments were performed in male C57BL/6 (C57) mice, which present a single renin gene [22], weighing 23 g on average, and that were bred and maintained in the Laboratory of Transgenes and Cardiovascular Control animal facility (Vitoria, ES, Brazil). The mice were fed a standard chow diet and provided water *ad libitum*. Animals were housed in individual plastic cages with controlled temperature (22°C) and humidity (60%) and were exposed to a 12 : 12 h light-dark cycle. All of the experimental procedures were performed in accordance with the National Institutes of Health (NIH) guidelines, and study protocols were previously approved by the Institutional Animal Care and Use Committee (CEUA-Emescam Protocol no. 010/2009).

### 2.2. Induction of 2K1C Renovascular Hypertension

We used a mouse model of 2K1C angiotensin-dependent hypertension, as previously described [3, 4, 23]. Briefly, the animals were anesthetized (ketamine/xylazine 91/9.1 mg/kg, *i.p.*) and kept on a heating pad that maintained the body temperature at 37°C to avoid hypothermia. The left renal artery was exposed through a retroperitoneal flank incision and was carefully isolated from the renal vein, nerves, and connective tissues. A U-shaped stainless steel clip with a 0.12 mm opening width was placed around the renal artery close to the abdominal aorta, which resulted in partial renal perfusion occlusion [24]. The wound was sutured, and the animal received a single injection of benzylpenicillin benzathine (7 mg/kg, *i.m.*) followed by recovery under care for 24 h. Control mice underwent the same surgical procedure except for the renal artery clip placement (Sham). 

### 2.3. Hemodynamic Measurements

Fourteen days after the renal artery clipping (2K1C) or Sham operations, the animals were anesthetized with a combination of ketamine/xylazine (91/9.1 mg/kg, *i.p.*) and the right common carotid artery was exposed and isolated through a cervical incision. A catheter (0.040 mm OD × 0.025 mm ID; Micro-Renathane; Braintree Scientific) was filled with heparin solution (50 UI/mL saline) and prior to insertion into the right carotid artery, which was subcutaneously tunneled and brought out at the nape of the neck. Immediately after surgery, animals received a single benzylpenicillin benzathine (7 mg/kg, *i.m.*) injection. The catheter was connected to a pressure transducer (Cobe Laboratories, USA), which was plugged into a pressure-processor amplifier and data acquisition system (MP100, Biopac Systems, USA) for mean arterial pressure (MAP) and heart rate (HR) recordings. After 48 hours, MAP and HR direct recordings were obtained while the animals were conscious and moving around freely in their cage.

### 2.4. Plasma Ang II Level Measurement

After hemodynamic measurements, blood was drawn through the arterial line into tubes containing EDTA and protease inhibitor cocktail (Product no. P2714, Sigma-Aldrich); the samples were centrifuged at 9.5 g for 15 min in a refrigerated centrifuge (4°C) to remove plasma for later analysis. Plasma Ang II was quantified by reverse phase high-performance liquid chromatography (HPLC). Briefly, peptides were initially separated in a reverse phase Aquapore ODS 300 column 7 *μ*m (4.6 × 250 mm) (Applied Biosciences, Foster City, CA, USA) using a linear mobile phase gradient from 5 to 35% (acetonitrile in 0.1% phosphoric acid) for 40 min using a 1.5 mL/min flow rate. Ang III (320 ng) was added to each sample as an internal standard, and the peptides were detected at 214 nm absorbance. Ang II was extracted using Sep-Pack-C18 column chromatography (Millipore, MA, USA) and was activated with 5 mL methanol, 5 mL tetrahydrofuran, 5 mL hexane, and 10 mL H_2_O (MilliQ). After activation, the samples were run through the column and eluted with ethanol : acetic acid : H_2_O (90 : 4 : 6, v/v). The last phase eluate containing Ang II was evaporated in a Speed Vac SC 110 (Savant Instruments, Holbrook, NY, USA) and reconstituted with 500 *μ*L 0.1% phosphoric acid in 5% acetonitrile, filtered, and injected onto the HPLC analytical column. Retention time was used to identify peaks of interest, which had been previously determined by standard peptide elution. The calculations were based on peak area, and Ang II concentration was expressed as pmol/mL blood.

### 2.5. Bone Marrow Mononuclear Cell Isolation

Mice were euthanized with a sodium thiopental overdose (100 mg/kg, *i.p*.) and marrow samples were collected from femurs and tibias that had been dissected and cleaned of all soft tissues. After removing the epiphyses and gaining access to the marrow cavities, whole BM was flushed out using a 26-gauge needle attached to a 1 mL syringe filled with Dulbecco's Modified Eagle Medium (DMEM; Sigma, St. Louis, MO, USA). MNCs were isolated by density-gradient centrifugation; the BM suspension in 4 mL DMEM was loaded on 4 mL Histopaque 1083 (Sigma-Aldrich) and centrifuged for 30 min at 400 g. The BM-MNC fraction was subsequently collected and washed in phosphate-buffered saline (PBS). A small volume of the resulting suspension was mixed with 0.4% trypan blue to perform cell count and viability analysis. Lymphocytes and undifferentiated cells were analyzed using a Neubauer chamber. 

### 2.6. DNA Damage Measurement with the Comet Assay

Bone marrow MNC DNA damage was analyzed by the alkaline comet assay as described by Singh et al. [25] with minor modifications. Regular microscope slides were precoated with 200 *μ*L 1.5% normal melting point agarose in distilled water, at 60°C (Sigma-Aldrich), dried overnight at room temperature, and then stored at 4°C until use. Subsequently, 2 × 10^4^ MNCs were mixed with 100 *μ*L 1% low melting point agarose in PBS at 37°C (Invitrogen, Spain) and spread on the agarose-coated slides using a coverslip. After gelling at 4°C for 20 min, coverslips were removed, and the slides were immersed in freshly prepared cold lysis solution (2.5 M NaCl, 100 mM EDTA, 10 mM Tris at pH 10–10.5, with freshly added 1% Triton X-100 and 10% DMSO) at 4°C for 1 h. After a 5 min wash in cold distilled water, the slides were placed in an electrophoresis chamber, which was then filled with fresh alkaline buffer (300 mM NaOH, 1 mM EDTA, pH > 13) for 20 min at 4°C. Electrophoresis was performed at 300 mA and 25 V for 30 min. All of these steps were conducted without direct light to prevent additional DNA damage. The slides were washed three times for 5 min with 0.4 M Tris buffer, pH 7.5, for neutralization. Finally, 100 *μ*L 20 *μ*g/mL of ethidium bromide (Sigma-Aldrich) was added to each slide, covered with a coverslip, and analyzed at a 20x magnification using a fluorescence microscope (Olympus BX60, United Kingdom) that had been equipped with excitation (510–550 nm) and barrier (590 nm) filters.

DNA damage was evaluated using visual classification of comets into five levels according to comet tail size from 0 (undamaged with no tail) to 4 (maximally damaged with long tail). The DNA damage extent was expressed in arbitrary units (a.u.). Three hundred randomly selected cells (100 cells from each of three replicate slides) were analyzed from each animal, and three AU values were generated for each animal, which were averaged to obtain the final result per animal. The group damage index (DI) ranged from 0, in which all of the cells were undamaged (300  cells × 0), to 1200, in which all of the cells were maximally damaged (300  cells × 4) [26]. The damage frequency (%) was calculated based on the number of cells with tails versus those without tails [27] and the % DNA damage was the fraction of each damage level relative to all of the comets that were analyzed.

### 2.7. Intracellular Superoxide Anion Fluorescence Measurement

Nonfluorescent dihydroethidium (DHE) was used for intracellular •O_2_^−^ detection by flow cytometry. Hydroethidine is freely cell permeable and is rapidly oxidized by superoxide to ethidium, which binds to DNA and amplifies the red fluorescence signal. To estimate the •O_2_^−^ content in the cell suspension, 10^5^ BM-MNCs were stained with 160 *μ*M DHE, followed by a 30 min incubation at 37°C in the dark to facilitate dye loading. DHE-loaded cells were treated with 10 mM H_2_O_2_ to oxidize the dye as a positive control. After 5 min of H_2_O_2_ treatment, the BM-MNCs were washed with PBS and cellular ROS levels were analyzed immediately with a FACSCanto II flow cytometer (Becton Dickinson, San Juan, CA, USA). Ten thousand events were recorded from each sample, and forward and side scatter gates were used to select single cells from clumps and debris. Specific fluorescence intensity was expressed as the median fluorescence intensity from the average of at least three repeated experiments in a.u. Red fluorescence was detected between 564 and 606 nm using a 585/42 bandpass filter. The Data were acquired and analyzed using BD FACSDiva software (BD). 

### 2.8. Statistical Analysis

The data are presented as representative figures or as the means ± SEM. The flow cytometry data are expressed as median fluorescence intensity (MFI) ± coefficient of variation (CV) of 3 repeated and statistically reproducible (Friedman test) measurements of at least five independent animals. Normality was evaluated using the Kolmogorov-Smirnov test. Statistical analysis was performed using Student's *t*-test for comparison of two independent groups, and two-way analysis of variance (Anova) followed by the Bonferroni's *post hoc* test was used for comparison of more than 2 groups. The Mann-Whitney test was used to compare the rank sum for the MFIs from the oxidative stress experiments. *P* values <0.05 were considered to be statistically significant.

## 3. Results

### 3.1. Body, Heart, and Kidney Weights, Blood Pressure, Heart Rate, and Plasma Ang II

Initial body weight was statistically similar among the groups. At the end of the experiments, body weight was reduced and ventricular weight was increased in the hypertensive group compared with the Sham group (Table 1). The hypertensive group also showed a significant increase in ventricular weight compared with the Sham group. Fourteen days after clip application, the left kidney was atrophic, while the right kidney displayed compensatory hypertrophy in the 2K1C mice.

**Figure 1:**
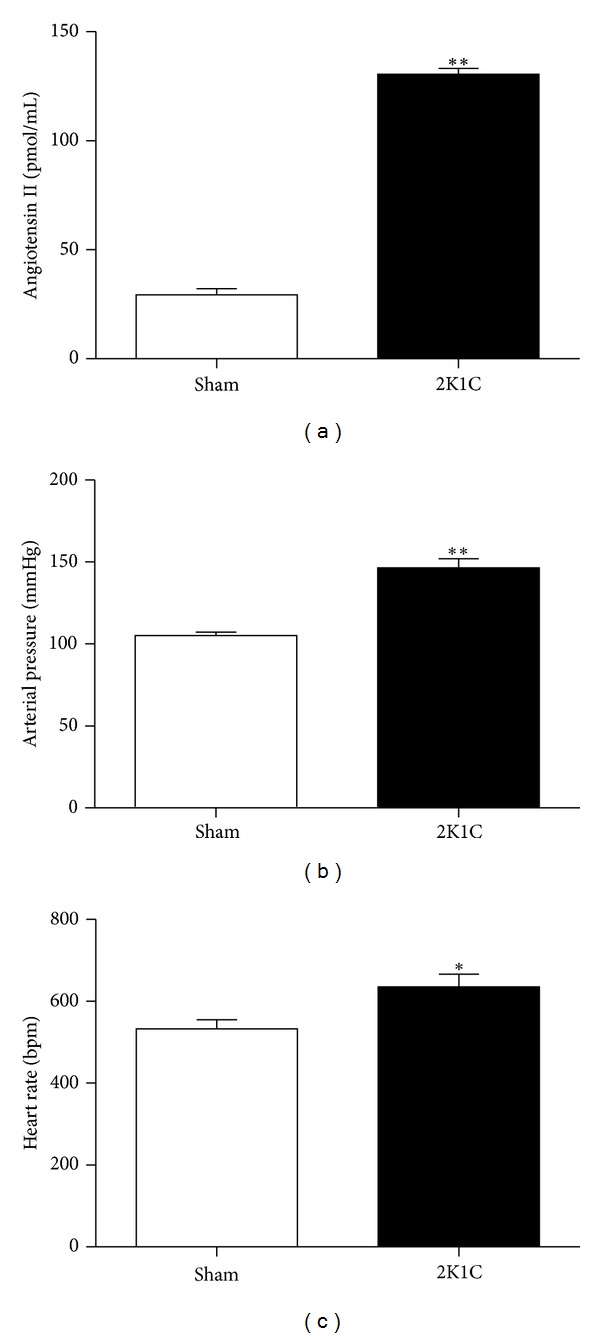
Bar graphs demonstrating resting mean arterial pressure, heart rate, and plasma angiotensin II values in conscious Sham (*n* = 5) and renovascular hypertensive (2K1C, *n* = 5) mice. Values are the means ± SEM. *P < 0.05 and **P < 0.01 versus the Sham group (Student's *t* test for independent samples).

Figure 1 shows the average values of direct resting MAP and HR measurements in conscious animals 14 days after renal artery clipping. As expected, MAP was 40% higher (*P* < 0.01) in the 2K1C than in the Sham mice. Hypertension in the 2K1C mice was accompanied by tachycardia when compared with the Sham mice. Plasma Ang II concentration was 4.5-fold greater (*P* < 0.01) in the 2K1C than in the Sham mice as measured by HPLC. 

### 3.2. Bone Marrow Mononuclear Cells Analysis

**Figure 2:**
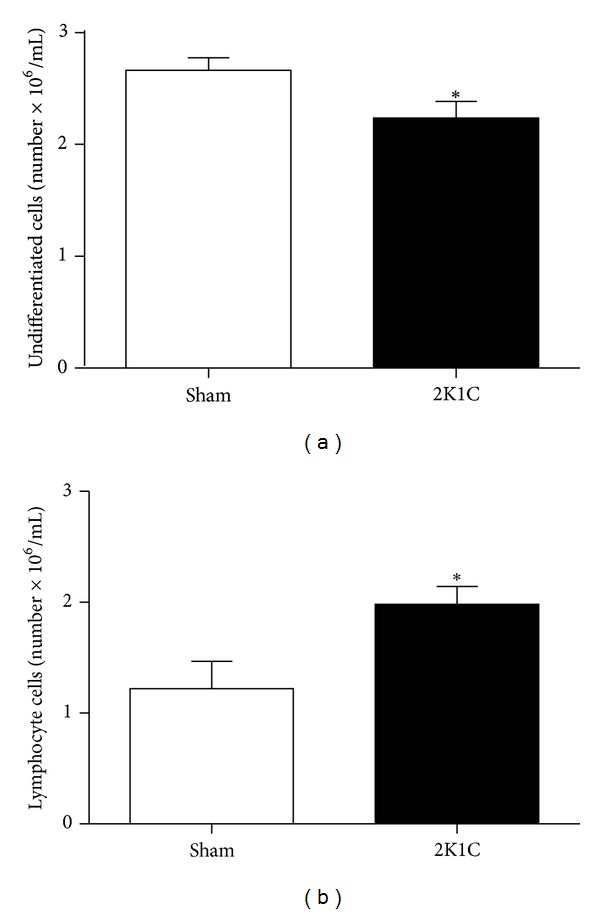
Neubauer chamber analysis of bone marrow mononuclear cells from Angiotensin-II-dependent hypertensive (2K1C, *n* = 10) and normotensive (Sham, *n* = 10) mice. Values of lymphocytes and undifferentiated cells are the means ± SEM. *P < 0.05  versus Sham group (Student's *t* test for independent samples).

We first assessed the effect of Ang II-dependent hypertension on BM-MNC viability and number using a Neubauer chamber after BM separation with a density gradient. Cell viability was assessed using the trypan blue exclusion method, and no differences were found between the groups (Sham: 97 ± 0.54% versus 2K1C: 96 ± 0.54%). To investigate whether the MNC number was reduced in the 2K1C mice compared with the Sham mice, we quantified lymphocytes and undifferentiated cells. As shown in Figure 2, the 2K1C mice had increased lymphocyte counts (62%) with a simultaneous reduction in undifferentiated cell number (18%) compared with the control animals. 

### 3.3. DNA Damage Measurement with the Comet Assay

**Figure 3:**
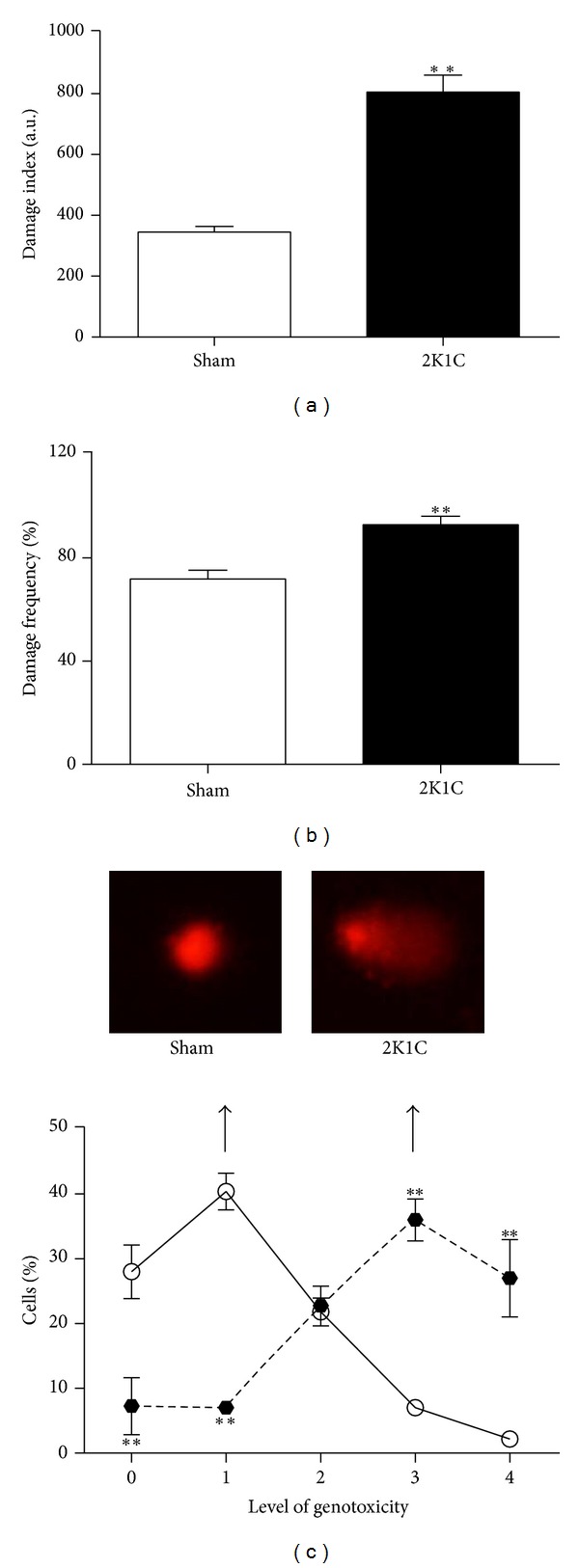
Bone marrow mononuclear cell DNA damage assessed by the comet assay. The left bar graphs demonstrate the average DNA damage index (a) and frequency (b). **P < 0.01 versus the Sham group (Student's *t* test for independent samples). Fluorescent images (c) are typical comets demonstrating increased DNA fragmentation in a renovascular hypertensive (2K1C) mouse compared with a normotensive (Sham) mouse. The lines graph (c) demonstrates the average percentages of DNA damage percentages for each genotoxicity level, comparing the 2K1C (filled circles, *n* = 5) with the Sham (empty circles, *n* = 6) mice. **P < 0.01 versus the Sham group (two-way Anova). Values are the means ± SEM.

The comet assay is a versatile and sensitive method for quantifying and analyzing DNA fragmentation in individual cells that can be used to assess oxidative DNA damage. The basic principle of the comet assay is DNA electrophoresis in an agarose matrix. Because the fragmented DNA migrates, the cells look like a comet under microscope with a head containing intact DNA and a tail containing DNA fragments [24]. Genomic DNA fragmentation incidence was visually analyzed according to comet appearance. To elucidate Ang II-dependent hypertension effects on BM-MNC, DNA damage was scored into five classes according to tail size and the relative tail DNA content indicates the amount of DNA damage. A significant predominance of low genotoxicity levels 0 and 1 in the Sham animals (level 0: 28 ± 3.4 and level 1: 40 ± 2.4%) compared with the 2K1C mice (level 0: 7 ± 4.4 and level 1: 7 ± 1%) was observed. In contrast, severe genotoxicity levels 3 and 4 prevailed in the 2K1C mice (level 3: 36 ± 3.2 and level 4: 27 ± 6%) compared with the Sham mice (level 3: 7 ± 0.4% and level 4: 2 ± 0.4%). Genotoxicity levels are demonstrated as typical images and average values in Figure 3. Moreover, DNA fragmentation was quantified using the DNA damage index and frequency (Figures 3(a) and 3(b)). The 2K1C mice had increased DNA damage as indicated by a higher damage index (Sham: 345 ± 19  versus 2K1C: 806 ± 55 a.u.) and frequency (Sham: 72 ± 3% versus 2K1C: 93 ± 4%). 

### 3.4. Ang II-Dependent Hypertension Induced ROS Production

**Figure 4:**
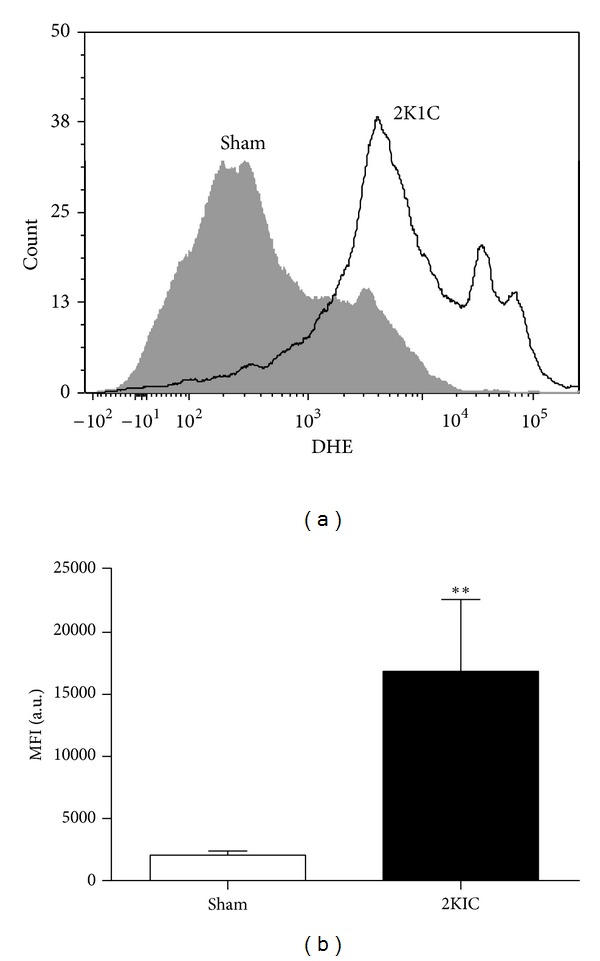
Effects of renovascular hypertension on oxidative stress in bone-marrow-mononuclear cells (BM-MNCs). (a) contains representative histograms of intracellular oxidation of dihydroethidium (DHE) to ethidium by BM-MNC from a hypertensive (2K1C) and a normotensive (Sham) mouse as evaluated by flow cytometry. The bar graph summarizes the median fluorescence intensity (MFI) values of DHE-loaded BM-MNC from 2K1C (*n* = 5) and Sham (*n* = 5) mice. Values are the medians ± coefficient of variation. **P < 0.01 versus the Sham group (Mann-Whitney test).

The above findings led us to further investigate the effects of Ang II-dependent hypertension on ROS production in BM-MNC. DHE is a membrane-permeable blue fluorescent dye that rapidly accumulates in the cytoplasm, where it is oxidized by •O_2_^−^, resulting in red nuclear fluorescence that can be measured by flow cytometry. In Figure 4, representative histograms (Figure 4(a)) and average (bar graphs) •O_2_^−^ production values are shown. As demonstrated by the right shift and in the bar graph, DHE median fluorescence intensity (MFI) values were significantly higher in the 2K1C than in the Sham group (16856 ± 5809  versus 2051 ± 336 a.u., *P* < 0.01), indicating increased intracellular BM-MNC oxidative stress.

## 4. Discussion

 The main finding of this study was marked DNA fragmentation in BM-MNC from Ang II-dependent hypertensive mice, most likely because of augmented •O_2_^−^ production and consequent oxidative stress. Further studies including other experimental models should be designed to discriminate the relative influence of hypertension and Ang II on this process. 

Renal artery clipping is accompanied by activation of the RAS and hemodynamic alterations [14, 28–30]. Higher levels of plasma renin and Ang II in 2K1C mice have been observed between 7 and 14 days after clipping and have returned to normal values by day 28 [14, 28, 30–32]. Based on these observations and on a previous publication from our laboratory [2], we performed this study two weeks after renal artery clipping. As expected, 2K1C mice showed atrophy of the clipped kidney and hypertrophy of the contralateral kidney. 2K1C mice exhibited high blood pressure levels accompanied by tachycardia, in agreement with previous studies [2, 5, 24]. In this study, we confirmed the high plasma Ang II, corroborating the concept that RAS activation plays a pivotal role in hypertension development in this murine model. In addition to the pressor and positive chronotropic effects, Ang II also stimulates cardiomyocyte protein synthesis [33–36], which in addition to hypertension may explain the cardiac hypertrophy that we observed in the 2K1C hypertensive mice. On the other hand, future studies should consider the measurement of protein levels of the ventricles as an index of hypertrophy. Taken together, these data suggest that the 2K1C mouse exhibits the main features of endogenous Ang II-dependent hypertension at this time point. 

 In addition to the systemic actions of the RAS, many tissues and organs have a local RAS, which can have paracrine, autocrine, and intracrine functions [37]. BM is the major reservoir for adult organ-specific stem cells, including endothelial progenitor cells (EPCs), hematopoietic stem cells (HSCs), and mesenchymal stem cells (MSCs). In this context, the presence of a complete local BM RAS that affects physiological and pathological blood cell production was hypothesized by Haznedaroglu et al. [38] and has recently been confirmed [9]. In our study, we found augmented lymphocytes and diminished numbers of undifferentiated BM cells in 2K1C hypertensive mice. Considering the presence of RAS components in HSCs [39] and stromal/MSCs [9], it is reasonable to propose that RAS may also be locally activated in BM of Ang II-dependent hypertensive mice. However, this possibility still needs to be confirmed by subsequent studies.

 Accumulating evidence suggests that the local RAS is actively involved in BM cells proliferation, differentiation, and death. Of note, Ang II affects the entire BM-MNC pool, such as EPCs [40], HSCs [41, 42], and MSCs [9]. As recently demonstrated, Ang II consistently decreases the number of cultured EPCs through activation of AT1 receptors and induction of apoptosis [40]. In addition and considering that 2K1C hypertensive mice exhibit endothelial dysfunction [23], Ang II could activate inflammatory cells or cytokine production, which may be responsible for cell recruitment in inflammation [43–47]. Moreover, this vasoactive peptide directly stimulates erythropoiesis by augmenting erythropoietin hormone production [48], which regulates erythrocyte differentiation [49], through AT1 receptors [8, 41]. Accordingly, da Cunha et al. [50] and Cassis et al. [48] reported that angiotensin converting enzyme (ACE) inhibitors and AT1 receptor antagonist treatments cause anemia, demonstrating hematopoietic side effects of RAS blockers and indicating that Ang II plays an important role in hematopoiesis. Taken together, experimental evidence suggests that Ang II exhibits important hematopoietic effects by stimulating erythroid, myeloid, and lymphoid differentiation, resulting in augmented lymphocyte number and simultaneously diminished undifferentiated cell number. 

 There is growing evidence that increased oxidative stress, which results in excessive ROS generation, plays a role in cardiovascular diseases including hypertension, as recently reviewed by us and others [24, 51–53]. Because there is a link between ROS and RAS signaling [53–55], a key mechanism by which Ang II influences heart and vessel function could be via its ability to activate ROS production [24, 56, 57]. We observed pronounced DHE MFI augmentation in BM-MNC in the 2K1C hypertensive mice compared with Sham normotensive mice. The relationship between oxidative stress and increased blood pressure has been reported in many hypertensive animal models, including the SHR [58], DOCA-salt [59], the 2K1C [14], and the 1K1C [60], which have excessive •O_2_^−^ production due to augmented NAD(P)H oxidase activity [61–64]. Interestingly, in the p47phox knockout mouse with concurrent 2K1C hypertension augmented ROS production occurs via expression of this NAD(P)H oxidase subunit [14]. This enzyme can be activated by hemodynamic forces and vasoactive agonists, for example, Ang II [65–67], which is a powerful vasoconstrictor involved in hypertension pathogenesis that uses ROS as an intracellular signaling mediator [66]. In addition, it seems that Ang II induces the increase of ROS production in EPCs and that this oxidative stress accounts for the Ang II-mediated reduction of EPC number, as this effect can be blocked by cotreatment with an antioxidant [40] and it increases gp91phox expression in EPCs, which may contribute to oxidative stress [68].

As discussed above, the role of Ang II goes beyond controlling circulatory homeostasis as discussed above in the impact of this peptide in ROS production, which is stimulated by NAD(P)H oxidase activation [69]. Recent experimental studies have shown that, at high concentrations, ROSs such as •O_2_^−^ are capable of direct protein and lipid oxidation, which causes DNA fragmentation [70]. DNA damage, which frequently occurs in cells exposed to oxidative stress [71], is a form of cellular injury that contributes significantly to the development and progression of cardiovascular disorders [24, 64, 72]. 

The comet assay has been used to determine DNA fragmentation in blood cells in murine models of spontaneous atherosclerosis [73, 74] and renovascular hypertension [24]. However, this is the first time that the comet assay has been used to assess DNA fragmentation in BM-MNC from 2K1C mice. Our results clearly demonstrated augmented DNA fragmentation in BM-MNC from the 2K1C mice compared with the Sham mice. Augmented DNA damage has also been demonstrated in other animal models of hypertension, including kidney cells from DOCA-salt rats [75] and mouse infused with Ang II [21]. Furthermore, DNA damage caused by ROS occurs more commonly in hypertensive than in normotensive patients and can be reduced by antioxidant drugs [76]. Of note, in the perfused mouse kidney, DNA damage was caused by Ang II, not by induced vasoconstriction, since another vasoconstrictor did not cause DNA damage [21]. Moreover, Ang II induces genomic damage in cultured kidney cells most likely via oxidative mechanisms, which can be prevented by AT1 receptor antagonists and by antioxidants [77]. The 2K1C mice had increased •O_2_^−^ production, which is a highly reactive and short-lived radical that is responsible for ROS generation and can interact with nearby molecules, such as DNA [21, 78, 79]; thus, our data suggest that ROS plays a key role by inducing DNA oxidative damage in this model of Ang II-dependent hypertension. 

In conclusion, we demonstrated that arterial hypertension induced by endogenous RAS activation by clipping the renal artery for two weeks (the 2K1C mouse model) results in a marked increase in ROS production with consequent BM-MNC DNA damage. We speculate that Ang II effects may be due to circulating and local BM RAS; therefore, both systems may play a crucial pathobiological role in the DNA damage observed in BM-MNC of 2K1C hypertensive mice. Taking into account that BM-derived cells are responsible of maintaining, generating, and replacing differentiated cells as a consequence of physiological cell turnover or tissue damage due to injury, the data obtained by this study suggested that comorbidities, specifically Ang II-dependent hypertension, have to be particularly considered if autologous transplantation is intended, since the donor tissue (i.e., bone marrow) might be altered in its functionality.

## Figures and Tables

**Table 1:** Body, ventricular, and kidney weights of 2K1C and Sham mice 14 days after renal artery clipping.

Parameters	Sham (5)	2K1C (5)
Body weight (g)	24 ± 0.5	22 ± 0.8*
Ventricular dry weight (mg)	25 ± 0.7	27 ± 0.7*
Clipped kidney dry weight (mg)	37 ± 1.4	25 ± 4.2*
Contralateral kidney dry weight (mg)	40 ± 2.0	44 ± 1.7
Clipped kidney weight/unclipped kidney weight (mg/mg)	0.94 ± 0.02	0.56 ± 0.09**

Values are the means ± SEM. *P < 0.05 and **P < 0.01 compared with the Sham animals (student's *t*-test for independent samples).
